# Gender Associated High Body Mass Index in Allergic Diseases

**DOI:** 10.3889/oamjms.2015.008

**Published:** 2014-12-25

**Authors:** Violeta Lokaj-Berisha, Besa Gacaferri-Lumezi, Ganimete Minci–Bejtullahu, Hatixhe Latifi-Pupovci, Natyra Karahoda–Gjurgjeala, Naser Berisha, Teuta Morina

**Affiliations:** 1 *Department of Physiology & Immunology, Faculty of Medicine, University of Prishtina, Prishtina, Republic of Kosovo*; 2 *Department of Obstetrics and Gynecology, University Clinical Center, Prishtina, Republic of Kosovo*; 3 *Faculty of Medicine, University of Prishtina, Prishtina, Republic of Kosovo*

**Keywords:** allergy, atopy, body mass index, gender, total IgE, Republic of Kosovo

## Abstract

**BACKGROUND:**

The increasing prevalence of allergic diseases and atopy is affected by sex, age and lifestyle factors. Obesity and excess weight are reported to be potential risk factors for atopy and specifically for asthma symptoms in children and adults.

**OBJECTIVE:**

To assess the relation between body mass index (BMI) and allergic diseases in patients of both genders, as well as association of BMI with atopy in healthy subjects.

**METHODS:**

BMI (kg/m^2^), skin-prick test and total serum immunoglobulin E levels were assessed in 139 subjects: 109 were patients with allergic diseases (M to F ratio was 51:58) and 30 were healthy controls (M to F ratio was 6:24).

**RESULTS:**

The study population was grouped into asthma, asthmarhinitis, rhinitis, Urticaria oreczema and controls by BMI and sex. Females with the highest BMI were in asthma and urticaria/eczema group. Males with the highest BMI were in asthmarhinitis and urticariaeczema group. High BMI was associated with atopy in both genders of healthy controls. High levels of total IgE were in male allergic patients.

**CONCLUSION:**

High BMI was associated with asthma in females, urticaria/eczema in both genders and atopy in both genders of healthy controls. Higher levels of total IgE were concluded in male patients.

## Introduction

The prevalence of allergic diseases in recent years is raising novel theories about causes and consequences. Genetic factors, hygiene hypothesis, interplay of environment factors along with obesity, are some of the most studied circumstances to be attributed to allergy. According to some studies, especially in last two decades, there is an increase in obesity among individuals; not only in industrialized countries but in developing ones too [1]. Also there is a link between obesity and allergy, based on other epidemiologic surveys, pointing out increased risk of asthmaand atopy among obese subjects [1, 2]. Even though there are still not fully explained mechanisms to be elucidated, some immune-modulator role of hormones and adipokines released from adipose tissue, have been described [2, 3]. It is well known that adipose tissue secretes many bioactive molecules (e.g. leptin and adiponectin) involved in energy metabolism, but there are other functions performed by them, some resembling cytokines and others interleukins [e.g. tumor necrosis factor alpha (TNFα) and interleukin 6 (IL-6) [2, 4].

Hence leptin, aside from being long term regulator of energy balance, is to be associated with inflammation regarding down regulation of regulatory T cells (Treg) and recruitment/activation of macrophages [4]. Serum levels of this hormone are proportionally correlated to adipose mass and furthermore increased leptin levels are reported among obese subjects with asthma compared to healthy controls, suggesting its inflammatory characteristics [5]. Similarly, adiponectin role in energy metabolism is well established, serum levels are inversely correlated with body fat percentage in adults and it has anti-inflammatory effect [6]. Down regulation of IL10 secretion from macrophages and adipocytes, has been proved in many animal studies and its anti-inflammatory effect is supported by lower levels in obese compared to non-obese children with asthma [3, 6]. Taken together these two adipokines are to change immunological mechanisms, resulting in hyper-reactive immune system, decreased immunological tolerance and shift to TH2 cytokine profile, which increases risk of obesity-associated autoimmune diseases and allergy [1, 6].

Several studies have emphasized negative relation between high body mass index (BMI) and bronchial reversibility in children with allergic rhinitis and asthma, positive association with prevalence of wheezing and eczema but no significant effect modification by either age or gender [7, 8]. However, some other studies reported no association between high BMI and doctor diagnosed or self reported asthma or allergy in young adults (age 26-29 year) [9, 10]. Yet, another survey demonstrated association between obesity and asthma (with or without allergic rhinitis) but not between obesity and allergic rhinitis only and pointed out that “The increased risk of asthma in young Swedish men with obesity has remained unchanged over a period of three decades” [1]. Another conflicting matter is association of IgE and BMI in allergic patients. Considering that high total IgE levels correlate with allergic diseases and prevalence of later ones correlate with high BMI, there is to be hypothesized an association between high total IgE levels and overweight or obesity in allergic patients as well as in atopic healthy controls with high BMI [12]. Nevertheless, several studies revealed the link between obesity and increased levels of total serum IgE in female patients with non-atopic respiratory allergies but no significant difference in serum total IgE levels according to BMI in male patients [13].

Eventually discrepancies exist among researchers about relation between total IgE, as marker of atopy, high BMI and allergic diseases, probably due to different ages of study population and specific outcomes to be expected; hence we decided to analyze these variables among our allergic patients and healthy controls with or without atopy.

## Material and Methods

### Subjects

This study included 139 subjects: 109 were randomly selected untreated outpatients who experienced allergy symptoms of asthma, rhinitis and skin allergies as acute urticariaoreczema (M to F ratio was 51:59) and were referred to the Allergy & Clinical Immunology outpatient service at the UCC Prishtina. We excluded patients with dermatographysm, and pregnant women. Thirty healthy volunteers, as control group, were medical staff (M to F ratio was 6:24) selected according to answers about not having, nor ever had, symptoms of respiratory allergies (coughing, sneezing, itching and nose discharge).

The patients were grouped according to their doctor diagnosed allergic disease; healthy volunteers according to their positive or negative reaction to skin prick test; all participants were grouped in obese and non-obese according to their body mass index (BMI), where BMI was classified as low (BMI, < 18.5 kg/m^2^), normal (18.5-24.9 kg/m^2^), overweight (25-29.9 kg/m^2^) and obesity (BMI, > or = 30 kg/m^2^), according to the World Health Organization (WHO), the US Preventive Services Task Force and the International Obesity Task Force.

### Study design

Informed written consent was obtained from all participants who were than included in the research. To each subject was handed over a study questionnaire requesting socio-demographic characteristics, including age and gender; height and weight were measured using a stadiometer and an electronic scale, respectively, under the supervision of the researchers; the BMI was calculated according to the body weight divided by the square of height (kg/m^2^); family history of atopy, respiratory symptoms and skin rashes/hives were recorded.

Protocol for the research project was approved by the Medical Faculty ethics committee.

### Blood sampling

For the measurement of total IgE, venous blood (2 mL) was obtained at 9:00 a.m. Blood samples were allowed to clot at room temperature and the serum was separated by centrifugation at 1,200×g for 10 minutes and stored at -80°C. Sera were thawed at room temperature before measurements. Total IgE levels were measured by RIA, using Immunotech Beckman Coulter company products (France) at the laboratory of Endocrinology, Institute of Physiology, UCC Prishtina. Bound radioactivity was calculated with Gamma counter and values more than 183 IU/ml were considered raised.

### Skin prick test

All participants were subjected to SPT with test kit G aeroallergens, Allergopharma product (Reinbeck Germany), along with positive control – histamine (1mg/ml) and negative – saline. Allergenic extracts included 4 groups of aeroallergens: pollens, house dust mites, animal dander and moulds. The allergenic extract was placed on to the volar surface of forearm and introduced into the epidermis with sterile lancet (1 mm depth), new for each allergen. For each subject, 15 minutes later, both diameters of skin reaction were recorded and SPT was considered positive if diameter >3 mm.

### Statistical analyses

Data are presented as the median and range. Kruskal−Wallis variance analysis was used for screening differences among the five groups. Fisher exact test was used to compare differences between two groups among healthy controls (with and without positive skin prick test). *P*−values less than 0.05 were considered significant.

## Results

The overall male to female ratio of one hundred thirty nine participants was 57:82. One hundred and nine subjects were patients with doctor diagnosed allergic diseases, as allergic rhinitis 60.5% (Rh), allergic asthma with rhinitis 18.35% (A/Rh), allergic asthma without rhinitis 7.3% (A) and urticariaoreczema 13.7% (U/Ecz). Among 30 healthy controls 37% reacted positive to at least one allergen on skin prick test.

The overall median age was 30.6 years (range 11-59, SD 11.9) with male (M) to female (F) ratio 26.9:33.1 years respectively, and according to diseases and age groups the median age in A group was 29.4 years (range 12-55), 29.1 years in A/Rh group (range 11-54), 28.5 years in Rh (range 11-59), 38.8 years in U/Ecz group (range 21-53) and 32.4 years in healthy volunteers (range 14-57) (As shown in Table 1).

**Table 1:** General characteristics of participants in relation to BMI and diseases.

	A n=8	A/Rh n=20	Rh n=66	U/Ecz n=15	Control n=30	P-value
**Gender N(%)**						

F	4 (50.0)	10 (50.0)	32 (48.5)	12 (80.0)	24 (80.0)	P>0.05
M	4 (50.0)	10 (50.0)	34 (51.5)	3 (20.0)	6 (20.0)	

Age yr. (Mean ± SD)	29.4 ± 18.0	29.1 ± 13.9	28.5 ± 10.7	38.8 ± 9.5	32.4 ± 11.1	P= 0.017

**BMI N (%)**						

Normal	5 (62.5)	12 (60.0)	41 (62.1)	7 (46.7)	17 (56.7)	
Overweight	2 (25.0)	8 (40.0)	22 (33.3)	4 (26.7)	12 (40.0)	
Obese	1 (12.5)	-	3 (4.6)	4 (26.7)	1 (3.3)	

A- asthma without rhinitis; A/Rh – asthma with rhinitis; Rh – rhinitis only; U/Ecz – urticaria/eczema; F- Female; M- Male; BMI- body mass index (normal= 18.5-24.9 kg/m^2^, overweight = 25-29.9 kg/m^2^, obese >30 kg/m^2^).

When calculating BMI, we found out that 44% of all cases were overweight and obese, especially urticaria/eczema group with 55.3%. Nevertheless we found the same percentage among healthy controls with positive skin prick test results (As shown in Table 2). After evaluating gender differences, we noticed that females with asthma and urticaria oreczema were 50% overweight and/or obese (BMI median 25.6 kg/m^2^ range 24.6-26.4 and 27.7 kg/m^2^ respectively) compared to other groups as well as controls.

**Table 2:** General characteristics of healthy controls in relation to skin prick test results, BMI, age and gender.

	Skin prick test		Control n=30

	Positive n=11	Negative n=19
**Gender N(%)**			

F	9 (81.8)	15 (78.9)	24 (80.0)
M	2 (18.2)	4 (21.1)	6 (20.0)

Age yr. (Mean ± SD)	31.8 ± 13.3	32.7 ± 10.0	32.4 ± 11.1

**BMI N(%)**			

Normal	5 (45.5)	12 (63.2)	17 (56.7)
Overweight	5 (45.5)	7 (36.8)	12 (40.0)
Obese	1 (9.0)	-	1 (3.3)

F- Female; M- Male; BMI- body mass index (normal= 18.5-24.9 kg/m^2^, overweight = 25-29.9 kg/m^2^, obese >30 kg/m^2^).

Nevertheless, males in A/Rh group were 50% overweight, 66.6% in urticariaor eczema group and 66.7% in healthy controls (As shown in Table 3). So after analyzing skin prick test positive and negative subjects among healthy controls, with a purpose to find any connection between atopy, gender and BMI, we found out that 55.6 % of skin prick test positive females and 50% of males were overweight compared to non atopic ones. Hence males were much more overweight compared to females among almost all disease groups, except for asthma group, and among healthy controls too.

**Table 3:** Average of BMI in patients, according to gender and diseases, and compared to controls.

	BMI	A (n=8)	A/Rh (n=20)	Rh (n=66)	Urticaria (n=15)	Controls (n=30)
	N	4	10	32	12	24

F	Median (range)	25.6	21.8	23.0	27.4	24.0
		(24.6-26.4)	(18-28)	(15.8-32.8)	(20-42)	(16.7-36.2)

	Normal	2 (50.0)	7 (70.0)	21 (65.6)	6 (50.0)	15 (62.5)
	Overweight	2 (50.0)	3 (30.0)	9 (28.1)	3 (25.0)	8 (33.3)
	Obese	-	-	2 (6.3)	3 (25.0)	1 (4.2)

M	N	4	10	34	3	6

	Median (range)	22.4	25	23.4	28.4	25
		(16.9-46.0)	(15.3-29.4)	(10.0-31.5)	(24.3-31.9)	(23.4-29.4)
				

	Normal	3 (75.0)	5 (50.0)	20 (58.8)	1 (33.3)	2 (33.3)
	Overweight	-	5 (50.0)	13 (38.2)	1 (33.3)	4 (66.7)
	Obese	1 (25.0)	-	1 (2.9)	1 (33.3)	-

A- asthma without rhinitis; A/Rh – asthma with rhinitis; Rh – rhinitis only; U/Ecz – urticaria/eczema; BMI- body mass index (normal= 18.5-24.9 kg/m^2^, overweight = 25-29.9 kg/m^2^, obese >30 kg/m^2^).

In respect to total IgE levels we found significant differences between subjects with allergies and healthy controls in both genders (As shown in Table 4); however in females the highest median value was among Rhinitis group and the lowest in urticaria oreczema group (Median 68.6 IU/ml range 4.9-354 and 14.3 IU/ml range 0.44-120.4 respectively) and also we found interesting results of total IgE levels among healthy female controls because those who were positive to skin prick test resulted with higher median value compared to non-atopic (Median 89.9 IU/ml range 11.1-174.5 and 15.4 IU/ml range 2.3-168.6 respectively).

**Table 4:** Association between diseases, BMI and serum total IgE levels in patients and healthy controls.

		A=8	A/Rh=20	Rh=66	Urt/ecz=15	Controls=30	
	**Females**	**n=4**	**n=10**	**n=32**	**n=12**	**n=24**	**P-value**

	Age yr. (Mean ± SD)	44.0 ± 13.2	30.0 ± 13.4	30.9 ± 10.4	40.0 ± 9.1	32.1 ± 10.8	P= .03

	Normal	2 (50.0)	7 (70.0)	21 (65.6)	6 (50.0)	15 (62.5)
	Overweight	2 (50.0)	3 (30.0)	9 (28.1)	3 (25.0)	8 (33.3)
	Obese	-	-	2 (6.3)	3 (25.0)	1 (4.2)

IgE IU/ml Median (Range)	Normal	24.7 (7.2 - 263.7)	49.5 (4.9 - 211.0)	68.6 (17.7 - 354.0)	25.3 (0.4 - 61.8)	25.8 (2.3 - 174)	P=.012
	Overweight	21.4 (18.7 - 24.5)	55.2 (39.3 - 300)	63.5 (4.9 - 182.3)	39.2 (6.8 - 71.6)	11.1 (3.1 - 94.0)	
	Obese	-	-	58.5 (44 - 73)	9.1 (5.7-120.4)	174.5 (174.5 - 174.5)	
	Total	21.4 (7.16 - 263.7)	49.6 (4.85 - 211)	68.6 (4.9 - 354)	14.3 (0.44 - 120.4)	22.15 (2.3 - 174.5)

	P-value	P>0.05	P>0.05	P>0.05	P>0.05	P>0.05	

	**Males**	**n=4**	**n=10**	**n=34**	**n=3**	**n=6**	

	Age yr. (Mean ± SD)	14.8 ± 2.8	28.2 ± 15.1	26.1 ± 10.6	34.0 ± 11.8	33.5 ± 13.2	P= .047

	Normal	3 (75.0)	5 (50.0)	20 (58.8)	1 (33.3)	2 (33.3)
	Overweight	-	5 (50.0)	13 (38.2)	1 (33.3)	4 (66.7)
	Obese	1 (25.0)	-	1 (2.9)	1 (33.3)	-

IgE IU/ml Median (Range)	Normal	68.9 (68.9-68.9)	239.4 (18-250.7)	67.3 (4.4-134.7)	161 (161-161)	20.2 (17.7-22.7)	P= .029
	Overweight	-	58.4 (14.8-227.3)	94.2 (18.7-257.7)	350 (350-350)	11.7 (1.5-93.2)	
	Obese	75 (75-75)	-	309 (309-309)	205 (205-205)	-	
	Total	300 (68.9 - 350)	228 (14.8 - 350)	83.9 (4.39 - 350)	350 (161 - 350)	16.9 (1.5 - 93.2)

	P-value	P>0.05	P>0.05	P>0.05	P>0.05	P>0.05	

A- asthma without rhinitis; A/Rh – asthma with rhinitis; Rh – rhinitis only; U/Ecz – urticaria/eczema; BMI- body mass index (normal= 18.5-24.9 kg/m^2^, overweight = 25-29.9 kg/m^2^ , obese >30 kg/m^2^).

Whereas males, being much more overweight than females, resulted with higher median level of total IgE in all disease groups, compared to controls and compared to female patients, particularly in urticaria/eczema male patients we found the highest median level of total IgE (350 IU/ml range 161-350) but not in rhinitis group were it was the lowest one (83.9 IU/ml range 4.39-350) (Table 4).

## Discussion

Currently there is a debate about association between overweight and allergic diseases, as well as overweight and atopy, because some studies support this association and others don’t [1, 2, 7, 9, 11]. This is probably due to different study designs, different ages of study population and sometimes very small sample size for assessing inquired association and providing conclusive results, as is the case with our study. The fact that we found association between overweight and asthma in females, but not in males could be because the gender is an important modifier of BMI-related asthma risk in adolescents as well as in adults, according to some studies performed in larger size samples [16-18]. This point out some documented influence of sex hormones that might affect asthma mechanisms, especially when it comes to estrogen and progesterone imbalance in females. Considering that progesterone up- regulate beta2 receptors, there have been hypothesis that obesity may reduce progesterone levels and hence reduces beta_2_adrenoreceptor function, which in turn will reduce bronchial smooth muscle relaxation. However we found no low levels of progesterone in asthma female subjects (data not published yet). On the other hand, estrogen may have dual effect on asthma and BMI was positively associated with plasma estrogen in postmenopausal women in some studies reveling immune-modulating effect of estrogen in dose dependent manner, matter that we should further evaluate [19]. The lack of association between BMI and asthma in males are similar to findings of Leenen et al. who reported that sex hormone alterations were associated with visceral fat accumulation in women but not in men [20]. However the men age of 14.8 years in our asthma males could also be the reason for the discrepancy between asthma groups, because Joseph et al. reported associations of BMI, hospitalization and missed school days for asthma in male adolescents only [21]. Eventually these facts once again confirm that asthma dominates in males in adolescence but reverses in adulthood being most abundant in females.

Although we found the strongest association between BMI and urticaria/eczema in both genders, similar to some studies conducted by Silverberg et al. in American adults and Lee et al. in Chinese adults, several other studies failed to support association between obesity and atopy in adults, such are those conducted by Flaxeder et al. and Jarvis et al. [11, 22-24]. Taken all together our data supports the link between overweight and eczema/urticaria, probably due to proinflamatory cytokines secreted in adipose tissue, or restricted physical activity, sedentary life and high energy intake in adults, including the idea of some other specific relationship between obesity and eczema/urticaria, but don’t establish the cause-effect relationship.

It is noteworthy that significant experimental evidence suggests causal relation between atopy and obesity in healthy women reporting high levels of 17ß-estradiol in obese compared to non-obese healthy women [25]. Our results also support these findings, because among healthy controls skin prick positive women were 55.6 % overweight/obese, compared to skin prick test negative ones, suggesting that the predisposition toward a humoral-mediated immune response could be linked to steroidogenic enzyme content of adipose tissue for estrogen synthesis [27].

Also, the fact that several studies have found association between allergic diseases and overweight females only is not supported in this study because our results reveal association of high BMI with A/Rh and ezema/urticaria in male subjects too [19, 20, 26]. Furthermore, when differentiating between skin prick test positive and negative healthy controls, we found higher BMI among atopic ones. This could be, partially, because of the higher BMI in almost all male subjects in this study compared to females, and could be supported by the fact that men have a lesser percentage of body fat than women for a given BMI so higher BMI within the same overweight/obese group could be the risk factor for allergic diseases and atopy in males [28].

Nevertheless, we found higher total IgE levels among male patients, within all disease groups, compared to female patients and especially healthy controls. These results are similar to some studies and in contrast to others [13, 14]. In a large cohort study in asthma patients, conducted by Borish et al., it was reported that mean total IgE levels were higher among children and adolescents compared to adults, and males have higher IgE levels than females [14]. This is also supported by our study within all disease groups, because mean total IgE level was very high in asthma male subjects being adolescents (mean age 14.8 years) compared to female adults (mean age 44 years), and also very high IgE levels were related to gender but not to age in other disease groups. The higher levels of total IgE in males were largely related to cigarette smoking in some studies but not in others so when adjusted for smoking status we found out that 25%-50% of females in the study and the control group reported smoking habits, compared to males who reported smoking habits only in A/Rh and Rh group and with lower percentage than females with the same disease [29, 30]. So High IgE levels are not related to cigarette smoking in our male subjects. The strongest association of total IgE, BMI and disease was determined for eczema/urticaria in males, probably due to higher overall levels of IgE in males [11, 15].

The major strength of this study is that all cases and controls were confirmed by allergen-specific skin prick tests to nineteen common allergens. Second, data on weight and height of both cases and controls were obtained through direct physical measurements, conducted by a physician, which should be more accurate and reliable than self-reported values. Third, none of the participants was treated with steroids or other hormonal therapy prior to involvement in this survey.

Limitation to our study is a lack of measures of adiposity other than BMI, which was used to define overweight and obesity, so we were not able to distinguish central from peripheral obesity. Furthermore the fact that women tend to have more fat mass and men more muscle mass may also contribute to apparently gender specific relation between obesity and asthma. Second, the sample size was relatively small for some atopic disorders, especially when assessing gender based association between allergic asthma and obesity and between genders in healthy controls. Therefore it is possible that obesity is truly associated with allergic asthma in males but we failed to detect such association because of a low statistical power of our data an d also because age unmatched male controls. Taken all together, further studies are needed to investigate the association between obesity and asthma in males. This study has confirmed that our male subjects with allergic diseases are more overweight compared to females with the same diseases, but overweight is associated with asthma in females only. High BMI is also associated with atopy in healthy females and males. Median of total IgE levels is higher in male patients within all allergic diseases in respect to females, but there are no significant differences between gender, BMI and total IgE levels. Also this study extended observations to prove associations of high BMI with symptoms of eczema/urticaria in both genders. The observations and findings reported from this study should by no means be neglected, while additional research with larger sample size is necessary to verify if weight loss would mitigate or prevent symptoms of urticaria/eczema and asthma.
